# Predicting Autonomous Shuttle Acceptance in Older Drivers Based on Technology Readiness/Use/Barriers, Life Space, Driving Habits, and Cognition

**DOI:** 10.3389/fneur.2021.798762

**Published:** 2021-12-02

**Authors:** Sherrilene Classen, Justin R. Mason, Seung Woo Hwangbo, Virginia Sisiopiku

**Affiliations:** ^1^Department of Occupational Therapy, College of Public Health and Health Professions, University of Florida, Gainesville, FL, United States; ^2^Department of Civil, Construction, and Environmental Engineering, School of Engineering, University of Alabama at Birmingham, Birmingham, AL, United States

**Keywords:** older drivers, predictors, acceptance, automated shuttle, barriers, executive function, cognition

## Abstract

Shared autonomous vehicle services (i. e., automated shuttles, AS) are being deployed globally and may improve older adults (>65 years old) mobility, independence, and participation in the community. However, AS must be user friendly and provide safety benefits if older drivers are to accept and adopt this technology. Current potential barriers to their acceptance of AS include a lack of trust in the systems and hesitation to adopt emerging technology. *Technology readiness, perceived ease of use, perceived barriers*, and *intention to use* the technology, are particularly important constructs to consider in older adults' acceptance and adoption practices of AS. Likewise, person factors, i.e., *age, life space mobility, driving habits*, and *cognition* predict driving safety among older drivers. However, we are not sure if and how these factors may also predict older adults' intention to use the AS. In the current study, we examined responses from 104 older drivers (*M*_age_ = 74.3, *SD*_age_ = 5.9) who completed the Automated Vehicle User Perception Survey (AVUPS) before and after riding in an on-road automated shuttle (EasyMile EZ10). The study participants also provided information through the Technology Readiness Index, Technology Acceptance Measure, Life Space Questionnaire, Driving Habits Questionnaire, Trail-making Test Part A and Part B (TMT A and TMT B). Older drivers' age, cognitive scores (i.e., TMT B), driving habits (i.e., crashes and/or citations, exposure, and difficulty of driving) and life space (i.e., how far older adults venture from their primary dwelling) were entered into four models to predict their acceptance of AVs—operationalized according to the subscales (i.e., *intention to use, perceived barriers*, and *well-being*) and the *total acceptance* score of the AVUPS. Next, a partial least squares structural equation model (PLS-SEM) elucidated the relationships between, *technology readiness, perceived ease of use, barriers to AV acceptance, life space, crashes and/or citations, driving exposure, driving difficulty, cognition*, and *intention to use* AS. The regression models indicated that neither age nor cognition (TMT B) significantly predicted older drivers' perceptions of AVs; but their self-reported *driving difficulty* (*p* = 0.019) predicted their *intention to use* AVs: *R*^2^ = 6.18%, *F* (2,101) = 4.554, *p* = 0.040. Therefore, *intention to use* was the dependent variable in the subsequent PLS-SEM. Findings from the PLS-SEM (*R*^2^ = 0.467) indicated the only statistically significant predictors of *intention to use* were *technology readiness* (β = *0.247, CI* = *0.087-0.411)* and *barriers to AV acceptance* (β = −*0.504, CI* = *0.285-0.692)*. These novel findings provide evidence suggesting that *technology readiness and barriers* must be better understood if older drivers are to accept and adopt AS.

## Introduction

Estimates indicate that older adults are the fastest growing segment of the population, and that they want to continue to drive, or stay mobile in their communities, if driving is no longer an option. Although many of them will continue to drive, we know that some of them are outliving their driving expectancy and need to retire from driving (1). Shared autonomous vehicles services (i.e., automated shuttles, AS) are being deployed globally and may improve older adults' (>65 years old) mobility, independence, and participation in the community, if they can no longer drive, choose not to drive, or if they are seeking to use alternative forms of transportation. However, AS must be easy to use, provide safety benefits, and instill trust if older drivers are to accept and adopt this technology. General barriers to older drivers' acceptance of AVs includes lack of trust in the systems, fear that driving abilities may decline due to relying on automation, and hesitation to adopting the technologies. Although research is emerging to inform us on the perceptions of older drivers pertaining to their acceptance practices, we are not certain how *demographics, technology readiness, ease of use* of the technology, and the *perceived barriers* related to the technology, may influence their *intention to use* such technology. Moreover, we also expect that a restricted *life space* and *driving history—*may further impact such *intention to use* practices. Finally, *cognitive* status may be a factor underlying older adults' *intention to use* AVs—especially if they need to retire from driving or if they can no longer drive. As such, this study examines how *age, technology readiness, perceived ease of use* of technology*, life space, driving habits*, and *cognition* predict acceptance (*intention to use*) of autonomous shuttles (AS). Understanding the singular and collective impact of such variables, will yield information that will inform city mangers on transportation planning practices for older adults, and assist industry partners with refining, designing, and deployment tactics targeted at older adults.

## Literature Review

### Older Drivers

Due to increased longevity, worldwide patterns are unfolding suggesting that 703 million persons aged 65-plus lived across the globe in 2019—and that by 2050, one in six people in the world will be over the age of 65 (2). Our aging population in the U.S. at 40.3 million in 2019, will account for one in four adults being 65-plus by 2030—or 80 million older adults in the U.S. (3).

Old age is associated with the onset of chronic conditions, comorbidities, frailty, and increased medication use (4). However, the aging population is a heterogeneous group (5–7) group and may include a mix of healthy and active older adults; people living with chronic disease; people with mild, moderate, or severe cognitive impairment; and people with, e.g., neurodegenerative or other diseases (8). The literature studies on age-related sensory, cognitive, and motor changes and their impact on driving, are very comprehensive and indicate that such underlying factors plausibly affect fitness to drive abilities of older drivers (9–11). Older adults who experience significant cognitive and/or physical declines may reduce or stop their driving (1), limit their out of home or life space activities (12), and consequently feel isolated while also experiencing deteriorating physical and mental health (13), and an impoverished quality of life (14).

### Older Drivers and Autonomous Shuttles

The AS—one mode in the family of shared mobility services (15) holds plausible opportunities to allow older drivers who require an alternative to automobile driving, to stay mobile in their communities. Particularly, the use of AS, may preserve independence in community mobility among the aging population with cognitive (and/or other) declines (16). Specific benefits of using AS are related to increased health and safety (e.g., crash prevention, driving stress reduction, increased mobility for those unable to drive); a green environment *via* emission reduction; progressive transportation and city planning; congestion mitigation; infrastructure development; and access to services, leisure, and employment opportunities (17–19). Interestingly, estimates indicate that the over 65-plus group will encompass approximately one third of the mobility marketplace by 2060, with the broader “Silver Economy” majorly contributing to new and related Autonomous Vehicles (AV) business models (17). However, AS must be easy to use and provide safety benefits if older drivers are to accept and adopt this technology (20).

Despite current barriers to older drivers accepting AS that include lack of trust in the systems (21, 22) and hesitation to use the emerging technology (23, 24), research indicates that their perceptions change, positively, after being exposed to an AS, operating at Level 4 of automation (15, 25). Some researchers have assessed user perceptions (alone) *via* survey (26–29), while others have reported on favorable passenger experiences in AS after riding it (30). For example, such riders were positive toward the low travel speeds, observing the shuttle's ability to detect objects (e.g., cyclist next to a shuttle), the control of the shuttle, and access to an emergency button in shuttle. In a recent study, researchers identified specific factors, i.e., using other modes of transportation (e.g., bicycle or public transit), miles driven by car, income, male gender, and living in urban areas, as positive predictors of older adults' perceptions to use autonomous driving features (31).

### Older Drivers' Acceptance and Adoption Practices of Technology

The literature indicates that four constructs are important to consider for older adults' acceptance and adoption practices pertaining to AV technology (32–36). These are: technology readiness, perceived ease of use, perceived barriers, and intention to use the technology, next discussed.

#### Technology Readiness

The Technology Readiness Index 2.0 [TRI; (32, 37)] is a measure determining optimism, innovativeness, discomfort, and insecurity of participants pertaining to new technologies on a 6-point scale, measuring the variables from 6 = very desirable to 1 = very undesirable. This multi-item scale yields acceptable psychometrics, and although not geared toward the older adults specifically, examines individual's readiness to use technology across the four categories (optimism, innovativeness, discomfort, and insecurity).

#### Perceived Ease of Use

This factor is contained within the Technology Acceptance Model [TAM; (33)]. The TAM, widely used in the literature to determine older adults' acceptance of information technology, explains about 40% of the variance in individuals' intention to use technology, and helps to understand user ease of use of the technology (34). Limitations, however, pertains to the TAM's lack of predicting cost, cultural differences, and social aspects of decision making in acceptance of such technology (35).

#### Perceived Barriers of AV Acceptance

The Autonomous Vehicle User Perception Survey [AVUPS; (36, 38)] contains three subscales (i.e., *intention to use, perceived barriers*, and *well-being*) and a total *acceptance* score. The AVUPS showed acceptable face validity and the mean content validity index was 96% (38). The total AVUPS scores for test-retest reliability (*N* = 84) were significantly and strongly correlated with excellent reliability (ρ = 0.76, *p* < 0.001, ICC = 0.95). The separate Mokken scale scores for test-retest were also significantly and strongly correlated with excellent reliability: i.e., *intention to use* (ρ = 0.80, *p* < 0.001, ICC = 0.93), *perceived barriers* (ρ = 0.73, *p* < 0.001, ICC = 0.87), and *well-being* (ρ = 0.72, *p* < 0.001, ICC = 0.84) (36). Because the construct validity indicated that either of the three separate Mokken subscales (i.e., intention to use, perceived barriers, and well-being), and/or the total acceptance score can be used to quantify users' perceptions of AVs (36), this tool may be used as a valid indicator for assessing older adults' perceived barriers, as well as their intention to use AVs.

### Person Factors as Predictors of AV Technology Acceptance

From the older driver literature, we know that person factors, i.e., age, life space mobility, driving habits, and cognition all predict driving safety among older drivers (6, 12, 39, 40). However—what is not known is if and how these factors will also predict older adults' intention to use the AS as a shared mobility service.

#### Life Space

Life space mobility indicates patterns of functional mobility that may change over time (6). Particularly, Stalvey et al., defines life space as the “spatial extent of an older person's mobility” (12). These researchers developed the Life Space Questionnaire (LSQ) as a reliable and valid measure to determine the mobility and independence of community-dwelling older populations over time. Life space mobility as a concept, is widely documented in the older driver literature, and is associated with personal, cognitive, functional, environmental, and social factors that affect how people live their day-to-day lives (6, 41). In a comprehensive review of the literature, conducted from 2010 to 2020, Johnson et al. (6) surmise that life space can be understood as an independent or dependent variable in older adults. Particularly, as an independent variable, life space is predictive of cognitive declines, admissions to nursing homes, falls, decreased quality of life, and mortality (42–45). Likewise, as a dependent variable, life space is associated with impairment in walking, various modes of transportation use, and car driving in older male and female adults (40, 46). It seems reasonable to surmise that a decline in life space mobility may lead to an increased desire to use the AS as a viable transportation option.

#### Driving Habits

Aging is associated with increased adoption of self-regulation strategies (e.g., limiting driving to only drive in optimal conditions, avoiding night driving or driving in traffic, or seeking alternative forms of mobility), driving fewer days per week, failing an on-road assessment, and unsafe driving such as observed in violations, crashes and/or citations, or driving cessation (7, 47–50). Such driving habits are generally assessed in the older driver literature via the Driving Habits Questionnaire [DHQ; (50)]. However, we do not know if declining driving habits, assessed by the DHQ, are associated with AS acceptance practices—and a general review of the literature yielded no findings to support (or not) this statement.

#### Cognition

Cognitive declines may lead to a deterioration in driving performance and essentially be a plausible factor underlying unsafe driving over time (51). According to researchers (39, 52, 53), cognitive predictors of older drivers failing an on-road evaluation, or being crash involved include: decreased visual attention [i.e., sustained, divided, selective, or switching attention (54)]; decreased visual processing speed [i.e., amount of time needed to make a correct judgment about a visual stimulus (55)]; decreased spatial abilities [i.e., generation, retention, retrieval, and transformation of visual-spatial information (56, 57)]; and decreased reaction time, [i.e., being able to respond quickly and carry out tasks concurrently (58)]. Moreover, impaired executive functioning [i.e., control and coordination of cognitive operations including planning, reasoning, problem solving, decision-making, judgement (59, 60)]—may lead to a degradation of driving tasks in older drivers (16). What is not clear from the current literature is if and how impaired cognitive abilities predictive of poor driving performance may also be telling of older adults' AV acceptance practices.

#### Summary

Although some of the aforementioned factors inform us on older driver perceptions pertaining to accepting AV technology, we are less informed about how these factors, combined with person factors, may be predictive of older adults' intention to use the AS as a viable mode of transportation.

### Rational and Significance

Our country and the world are aging. Yet, the desire to stay mobile and to participate in their communities are paramount among older adults. Age-related declines are affecting the safety and fitness to drive abilities of older drivers which eventually impair their independence in community mobility and participation in society. Although autonomous vehicle technologies, specifically the AS, a shared mobility service, holds plausible community mobility opportunities for older adults, we do not yet understand the effect of *age, technology readiness/use/barriers, life space mobility, driving habits*, and *cogniti*on—as singular or cumulative predictors of intending to use such technology.

### Assumptions

Based on the literature, and our past and current findings on older drivers' acceptance practices of AS, we have formulated four assumptions: (1) older age (vs. younger age) will be a barrier of AS acceptance; (2) decreased cognitive status will be a barrier in AS acceptance; (3) driving habits (i.e., increased driving difficulty, crashes and/or citations) will positively predict AS acceptance; and (4) decreased life space mobility will positively predict AS acceptance. Finally, we anticipated that the predictor variables will singularly or cumulatively explain the eventual acceptance and adoption practices of older drivers—and hence we developed a conceptual model to explore the multi-variate relationships.

### Purpose

The primary purpose of this paper is to examine if *age, technology readiness/use/barriers, life space, driving habits*, and *cogniti*on are predictors of older adults' *intention to use the technology*. This information is critical to help inform city managers and transportation planners as they develop AS deployment practices. Likewise, findings will be very relevant to industry partners, who must refine design factors, to provide ubiquitous access and acceptability to older adults if they are to use the AS.

## Methods

The University's Institutional Review Board (IRB#201801988) provided approval for the study and all participants consented to enroll and participate in the study. Participants received $25.00 upon completing the study.

### Design

This is a secondary analysis from a pre-posttest experimental design study (15). For this study we utilized surveys at baseline and after exposure to the automated shuttle (AS). We enrolled participants who were recruited from community partner interactions, older driver stakeholders, flyers placed in community settings, and Facebook groups. Detailed methodology and research protocol are discussed in our previous publications (15, 36, 38).

Community-dwelling older drivers (*N* = 104) were included in the parent study if they were 65 years of age or older, had a valid drivers' license, and had driven in the last 6 months. They were excluded if they scored <18 Montreal Cognitive Assessment (MoCA) or were unable to communicate in English. In this study, older drivers were relatively independent as the eligibility criteria reduced heterogeneity of our sample by excluding individuals that displayed signs of impaired cognition, required routine assistance, and no longer maintained a valid drivers' license or driving exposure.

### Equipment

**Figure 1:**
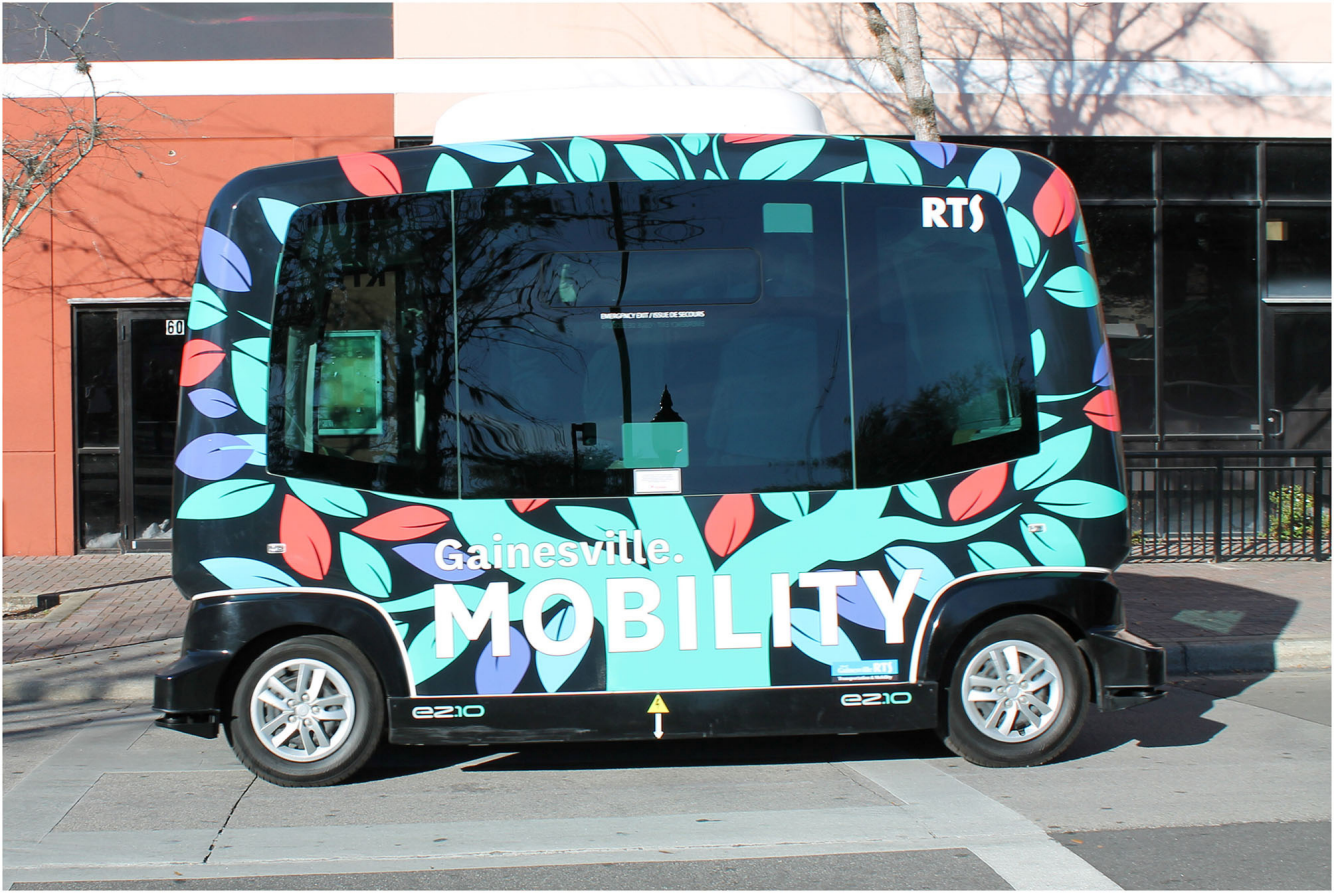
The EasyMile EZ10 automated shuttle (SAE Level 4).

The EasyMile EZ10 automated shuttle (SAE Level 4) operated with a safety operator in the vehicle, on a pre-designated route in a deserted bus depot (see Figure 1). The deserted bus depot was located in an urban environment next to a park, restaurants, and a new bus depot with various forms of transportation. The AS operated at roughly 15 miles per hour without the presence of ambient traffic or pedestrians. The AS ride was about 10 min in duration, between the hours of 9 AM and 4 PM, in an area with no traffic, bicyclists, or pedestrians, and in good weather conditions. Initially six participants were allowed in the shuttle, but due to COVID-19, we accommodated two participants in the shuttle. All the participants and research team adhered to CDC guidelines for COVID-19 prevention.

### Procedure

The detailed study protocol is available from Classen et al. (25). We are only discussing the procedure relevant to this analysis. As such, during the first visit, participants completed a demographic medical history form (61), TRI 2.0 (37), TAM (33), Automated Vehicle User Perception Survey [AVUPS; (36, 38)], Life Space Questionnaire [LSQ: (12)], DHQ (50), and the TMT A and B (62). Prior to riding in the AS, participants were instructed to remain seated while the shuttle operated. During the route, the safety operator detailed capabilities and features of the AS. The AVUPS was completed again during their final visit, i.e., after being exposed to both the autonomous shuttle and simulator. (Note, the simulator data are not analyzed in this study.)

### Measures

**Independent variables** for the exploratory path model included the following:

*Age*. The only variable used from the demographic medical history form for this analysis was age.

*Technology Readiness*. Four items were used from the Technology Readiness Index 2.0 [TRI; (37)], representing the validated domain, *optimism* (see Table 1).

**Table 1:** Items, item factor loading, internal consistency (α), average variance extracted (AVE), and construct reliability (CR) for the PLS-SEM (*N* = 104).

**Constructs/items**	**Item**	**λ**	**α**	**AVE**	**CR**
Technology Readiness			0.791	0.614	0.863
TRI 1	New technologies contribute to a better quality of life	0.846			
TRI 2	Technology gives me more freedom of mobility	0.777			
TRI 3	Technology gives people more control over their daily lives	0.821			
TRI 4	Technology makes me more productive in my personal life	0.680			
Perceived ease of use			0.736	0.555	0.831
TAM 7	My interaction with the autonomous vehicle is clear and understandable.	0.822			
TAM 8	Interacting with the autonomous vehicle does not require a lot of my mental effort.	0.579			
TAM 9	I find the autonomous vehicle to be easy to use.	0.798			
TAM 10	I find it easy to get the autonomous vehicle to do what I want it to do.	0.756			
Barriers to AV acceptance			0.780	0.532	0.790
AVUPS 5	I am suspicious of automated vehicles	0.679			
AVUPS 14	It will require a lot of effort to figure out how to use an automated vehicle	0.678			
AVUPS 16	I would rarely use an automated vehicle	0.722			
AVUPS 19^×^	My driving abilities will decline due to relying on an automated vehicle	<0.05			
AVUPS 26	I believe that automated vehicles will increase the number of crashes	0.734			
AVUPS 28	I feel hesitant about using an automated vehicle	0.825			
Intention to use			0.917	0.554	0.931
AVUPS 4	I am open to the idea of using automated vehicles	0.700			
AVUPS 6	I believe I can trust automated vehicles	0.683			
AVUPS 7^×^	I will engage in other tasks while riding in an automated vehicle	< .05			
AVUPS 8	I believe automated vehicles will reduce traffic congestion	0.759			
AVUPS 9	I believe automated vehicles will assist with parking	0.703			
AVUPS 13	I expect that automated vehicles will be easy to use	0.782			
AVUPS 15	I would use an automated vehicle on a daily basis	0.551			
AVUPS 17^×^	Even if I had access to an automated vehicle, I would still want to drive myself	<0.05			
AVUPS 20	I will be willing to pay more for an automated vehicle compared to what I would pay for a traditional car	0.585			
AVUPS 21	If cost was not an issue, I would use an automated vehicle	0.840			
AVUPS 22	I would use an automated vehicle if National Highway Traffic Safety Administration (NHTSA) deems them as being safe	0.868			
AVUPS 25	When I'm riding in an automated vehicle, other road users will be safe	0.813			
AVUPS 27	I feel safe riding in an automated vehicle	0.833			

*λ, Item Factor Loading (Criteria: > 0.5)*;

× *item was removed due to poor factor loading; PLS-SEM, Partial least squares structural equation modeling; TRI, Technology Readiness Index; TAM, Technology Acceptance Model; AVUPS, Autonomous Vehicle User Perception Survey. α, Cronbach's alpha; AVE, Average Variance Extracted; CR, Construct Reliability. Items for the Barriers of AV Acceptance construct are from the AVUPS Perceived Barrier scale*.

*Perceived Ease of Use*. Four items were used from the Technology Acceptance Model [TAM; (33)], representing the validated domain, *perceived ease of use* (see Table 1).

*Perceived Barriers to AV Acceptance. Six* items were used from the *perceived barriers*, a sub-scale of the AVUPS (36). The items and their loadings are indicated in Table 1.

*Life Space Questionnaire* [LSQ: (12)]. The LSQ, is a valid and reliable measure to ascertain how far older adults venture from their primary dwelling. The LSQ assesses mobility *via* nine space-levels (bedroom/sleep area, external area of the residence, yard/driveway, community, neighborhood, town, county, state, southeast region) accessed in the prior week. Each space level is scored according to the space reached (binary) which is represented by the nine LSQ items. The total score, obtained by summing the score on each level (i.e., each item), ranges from 0 (older adult restricted to the bedroom/sleeping area) to 9 (older adult traveled outside of southeast region). Participants were informed that their study visit should not influence their LSQ responses.

*Driving Habits Questionnaire* [DHQ; (50)]. The DHQ contains 34 items comprised of six factors, including *self-reported crashes and/or citations, driving exposure, driving space, current driving status, driving dependence*, and *driving difficulty*. The *self-reported crashes and/or citations* and *driving space* items are answered yes (1) or no (0). *Driving exposure* indicates the number of self-reported miles driven in the past year. *Driving space* reflects six space-levels (immediate neighborhood, beyond neighborhood, neighboring towns, distant towns, outside the state of residence, outside the region). Current *driving status* was used as a manipulation check for the inclusion criterion, i.e., “driving within the last 6 months with a valid driver's license” and the *dependence on other drivers*, ranges from 1 (“I drive”) to 3 (“this person drives me”). *Driving difficulty* (eight items) ranges from 1 (“so difficult I no longer drive in the situation”) to 5 (“no difficulty”) on a 100-point scale. The mean score of the eight-items is subtracted by 1 and multiplied by 25. A score below 90 suggests driving difficulty. The three factors used for this analysis was *self-reported crashes and/or citations, driving exposure*, and *driving difficulty*.

*Cognition: Trail Making Test Part A and Part B [TMT A and TMT B;* *(**62**)]*. TMT A and B are extensively used among researchers to assess executive functions, visual–perceptual functions and visual–motor tracking of older drivers (5, 47, 63, 64). TMT A requires participants to connect numbers and involves visual scanning, number recognition, numeric sequencing, and motor speed. Trails B requires participants to connect numbers with letters, alternating between the two sequences and measuring set shifting and mental flexibility. TMT B, a proxy variable for executive functioning (subtracting TMT A from TMT B), is a predictor of on-road performance in community-dwelling older licensed drivers (65).

### Dependent Variable

#### Intention to Use

The AVUPS contains *intention to use* as one of the sub-scales that demonstrated excellent reliability and validity (36, 38). The 13 items used in the *intention to use* subscale, are indicated in Table 1.

### Data Analysis

Descriptive statistics were conducted for participants' age and sex. Continuous data were presented as mean (*M*) and standard deviation (*SD*). Categorical data were presented as count (*n*) and percentage (%).

A series of multiple linear regressions, with backward stepwise selection, were conducted to predict the outcome variables, three AVUPS subscales and the total AVUPS acceptance score. The post-exposure AVUPS scores were used as our dependent variables. The best model for each outcome variable was selected based on simplicity and Akaike information criterion (AIC). The independence of residuals was assessed via a Durbin-Watson test. The linearity was assessed *via* partial regression plots and a plot of studentized residuals against the predicted values. Multicollinearity and collinearity were assessed using bivariate correlations and comparison of tolerance values and variance inflation factors [>2; (66)]. The final model was cross-validated using k-fold cross validation. The predictors for all four models included age (continuous), TMT B (continuous), four domains from the driving habits questionnaire: i.e., [driving dependence (ordinal), driving exposure (continuous), driving difficulty (continuous), crashes and/or citations (binary; no vs. yes)], and life space (ordinal). MoCA scores were not entered as predictors into our models as they were used as an exclusion criterion for participant selection. The AVUPS scores were assessed for normality *via* visual examination (i.e., histograms and probability plots) and statistical tests (i.e., Fisher's skewness, kurtosis, and Shapiro-Wilk test). The *p*-values were adjusted to control for multiple comparisons using the Benjamini-Hochberg procedure (67). A *p-value* of <0.05 was considered significant. Data were analyzed in RStudio (68) using R version 4.0.4 (69) and the tidyverse ecosystem (70).

**Figure 2:**
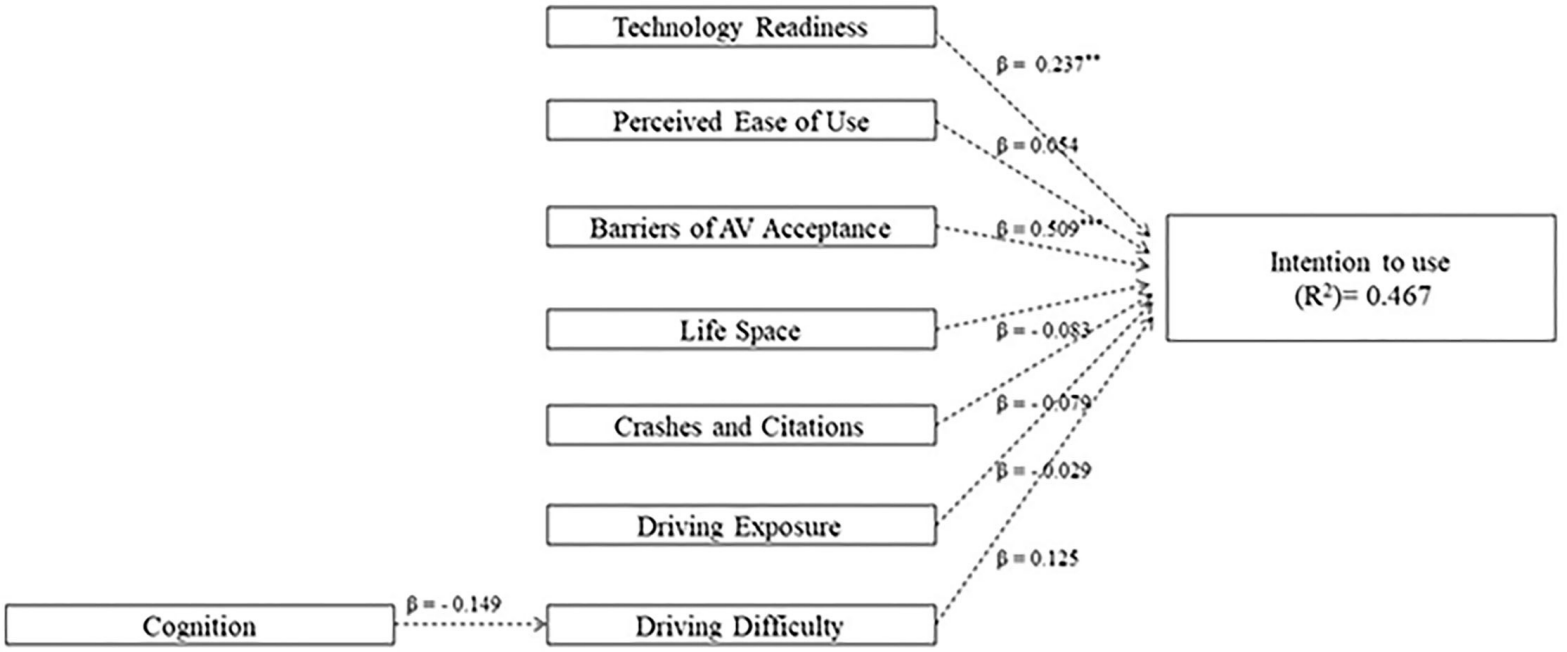
Bootstrapped pathway analysis predicting older adults' intention to use the technology.

An exploratory path model was formulated to elucidate the relationships between, *age, technology readiness, perceived ease of use, barriers to AV acceptance, life space, driving habits*, and *cogniti*on to *intention to use*. Specifically, PLS-SEM was deployed using SEMinR software (71). All scores were used from participants' baseline intake (i.e., pre-exposure) other than *intention to use*, which was collected after riding in the AS. The exploratory path model (Figure 2) displays the hypothesized relationships between *technology readiness* (TRI optimism domain), *perceived ease of use* (TAM domain), *barriers of AV acceptance* (AVUPS subscale)*, life space* (LSQ total score), *crashes and*/or *citations* (DHQ), *driving exposure* (DHQ driving exposure domain), *driving difficulty* (DHQ driving difficulty domain), *cognition* (Time to complete Trails B) to *intention to use* (AVUPS intention to use subscale). PLS-SEM was used due to the exploratory nature of our study, relatively small sample size, and its ability to builds upon multiple regression to investigate complex relationships between dependent and independent variables (72, 73). All scores entered into the PLS-SEM were normalized using the Blom transformation (74, 75), the most commonly deployed rank-based inverse normal transformation. Criteria used to evaluate the constructs were as follows: the (a) factor loading coefficients must be >0.5, (b) average variance explained (AVE) in each construct must be >0.5, and (c) composite reliability of each construct must be >0.7 (73). The structural model was evaluated by interpreting the magnitude of each path coefficients (β). The 95% Confidence Interval (CI) of each path coefficient was estimated by bootstrapping using the Monte Carlo method, whereby 5,000 random sub-samples were drawn with replacement from the item scores.

## Results

The study sample (*N* = 104 older drivers; *M*_*age*_ = 74.3 ± 6.0) was predominantly White (*n* = 93; 89%) and consisted of 47 (45%) males and 57 (55%) females, ranging from 65 to 91 years old. The three AVUPS domain scores and *total acceptance* score did not differ between genders (binary) (15); thus, gender was not used as a covariate in the models. Descriptive statistics for participants' *age, driving habits*, and *cognition* are displayed in Tables 2, 3.

**Table 2:** Indicators of continuous independent variables: Age, cognition, and self-reported driving habits (*N* = 104).

**Variables**	***M***	***IQR***	***SD***	**Range (min-max)**
Age (years)	74.30	70-78	5.95	65-91
TMT B (s)	78.66	50-91	41.26	29-257
MoCA score	26.91	25-29	2.23	21-30
Driving exposure^*^ (DHQ domain: miles/year)	6657.5	2,158-7,930	6694.7	208-35,360
Driving difficulty^*^ (DHQ domain)	81.21	75-91	15.24	16-100

* *DHQ domain; DHQ, Driving Habits Questionnaire; IQR, Inter quartile range; M, Mean; min, minimum; max, maximum; MoCA, Montreal Cognitive Assessment; SD, standard deviation; s, seconds; TMT B, Trail Making Test Part B*.

**Table 3:** Indicators of categorical independent variables: Driving dependence, driving space, and crashes and/or citations (*N* = 104).

**Variables**	***N* (%)**
**Driving dependence (DHQ domain)**	
“I drive”	47 (45%)
“Split between being driver and passenger”	40 (38%)
“This person drives me”	17 (16%)
**Driving space (DHQ domain)**	
Immediate neighborhood	0 (0%)
Outside neighborhood	6 (6%)
Neighboring towns	13 (12.5%)
Distant towns	39 (37.5%)
Outside of Florida	15 (14%)
Outside of southeast region	31 (30%)
**Crashes and/or citations (DHQ domain)**	
Yes	18 (17%)
No	86 (83%)

*DHQ, Driving Habits Questionnaire*.

Four multiple linear regression models with backward stepwise selection were conducted to predict AVUPS subscales (i.e., *intention to use, perceived barriers*, and *well-being*) and the *total acceptance* score. Histograms displayed negatively skewed AVUPS scores and difficulty with driving (i.e., DHQ domain) which were normalized using a reflect and square root transformation. For the first model, older drivers' self-reported *driving difficulty* (*p* = 0.019) and *crash and/or citation* involvement (*p* > 0.05) predicted their *intention to use* AS: *R*^2^ = 6.18%, *F*_(2,101)_ = 4.554, *p* = 0.040 (see Table 3). For the second model, *dependence on other drivers* (*p* = 0.052): *R*^2^ = 3.67%, *F* (1,102) = 3.875, *p* = 0.052 did not predict older drivers' *perceived barriers* to AS. For the third model, involvement in a *crash or citation* (*p* = 0.072): *R*^2^ = 2.18%, *F*_(1,102)_ = 3.297, *p* = 0.072 did not predict older drivers' *well-being* related to AS acceptance. Lastly in the final model, *driving difficulty* (*p* = 0.081): *R*^2^ = 1.99%, *F*_(2,101)_ = 3.101, *p* = 0.081 did not predict older drivers' *acceptance of AS*.

Discriminant validity was assured by the factor loading coefficients of each individual indicator used to identify *technology readiness* (0.680-0.846), *perceived ease of use* (0.579-0.822), *barriers of AV acceptance* (0.678-0.825), and *intention to use* (0.551-0.868) were consistently >0.5 (see Table 1). To meet this criteria, 2 of 13 items were removed from *intention to use* and one of six items was removed from *perceived barriers*. Convergent validity was assured by exceeding criteria for average variance extracted (AVE > 0.5), internal consistency (α > 0.7) and composite reliability (>0.7) for each construct (i.e., *technology readiness, perceived ease of use, barriers of AS acceptance, and intention to use)* with multiple indicators (see Table 1). Figure 2 displays the bootstrapped model, including the β coefficients, effect size (*R*^2^), loading coefficients (criteria: >0.5) for each construct. The effect size (*R*^2^ = 0.467) indicates that 47% of the variance in *intention to use* was accounted for by the predictors. Table 4 displays the significance of the path coefficients in Figure 2.

**Table 4:** Statistical significance of path coefficients in the structural bootstrapped model (*N* = 104).

**Path**	**Effect (*β)***	**Confidence Interval (95%)**	***t*-statistic**
Technology readiness to intention to use	0.247^**^	0.087-0.411	2.875
Perceived ease of use to intention to use	0.070	−0.129-0.288	0.511
Barriers to AV acceptance to intention to use	−0.504^***^	0.285-0.692	4.967
Life space to intention to use	−0.085	−0.241-0.064	1.102
Crashes and/or citations to intention to use	−0.069	−0.191-0.064	1.199
Driving exposure to intention to use	−0.031	−0.208-0.153	0.317
Driving difficulty to intention to use	0.126	−0.040-0.292	1.485
Cognition to driving difficulty	−0.151	−0.341-0.054	1.475

*β, path coefficient*;

** *p < 0.01*,

*** *p < 0.001*.

Table 4 indicates the statistical significance of the path coefficients in the structural bootstrapped model. From this model the only statistically significant differences occurred between *technology readiness* and *intention to use to use;* and *barriers to AV acceptance* and *intention to use*.

## Discussion

The primary purpose of this paper is to examine if *technology readiness, ease of use, technology barriers, life space mobility, driving habits*, and *cogniti*on are predictors of older adults' *intention to use* the AS.

Based on the literature, and our past and current findings on older drivers' acceptance practices of AS, we have formulated and tested four assumptions. The first assumption postulated that older *age* (vs. younger age) will be a barrier of AS acceptance—and this did not hold true. No obvious differences were observed between age (or genders) for AV acceptance despite the age range among the older adults with a spread from 65 to 91 years of age.

The second assumption postulated that decreased *cognition* will be a barrier in AS acceptance—which also was not supported by the findings. The MoCA score (*M* = 26.91, *SD* = 2.23) indicated that overall, the general cognition of the group was reasonably intact, and as such we did not detect a wide range in cognitive functioning, even given that the MoCA score of <18 was used as an exclusion criterion. The TMT B score (*M* = 78.66, *SD* = 41.26) indicates that the group had on average a faster completion time of the test (cut-off 180 s)—which is also better than the reported TMT B scores (108 s) with a statistically significant area under the curve of 0.86 to predict on-road failure in people with Parkinson's (76). However, wide variability (*SD* = 41.26) was noted in the TMT B scores of the older adults, suggesting that at least some of them were very likely to have had lower cognitive functioning. Yet, at least in our sample, cognition was not a predictor of the *intention to use* practices of older drivers.

The third assumption postulated that *driving habits* (i.e., crashes and/or citations, driving exposure, and increased driving difficulty) will positively predict *intention to use*. Just under 20% of the group had evidence of self-reported crashes and/or citations—yet this variable did not predict *intention to use*. Although projections from Lyman et al. indicate that future crash counts are hard to predict, they propose evidence indicating that older drivers will make up a substantially larger proportion of drivers involved in crashes (77), partly due to their increasing age, driving exposure, and need to continue to drive. Of course, a necessary mitigation strategy for avoiding crash risk is to suggest the use of an AS as a safer mode of transportation—but, it is clear that being crash and/or citation involved did not predict *intention to use* in our study. Likewise, even though we observed a big spread in miles driven per year (208-35,360) the older adults' exposure did not predict *intention to use*. Although the driving difficulty score (*M* = 81.21, *SD* = 15.24), slightly below the criterion of 90, suggests that some may have experienced a decline in fitness to drive abilities—this variable also did not predict the older adults' *intention to use*. From these findings, at least as they pertain to our sample, we learn that *driving habits* does not predict acceptance practices and as such, should not be used in such a fashion in future research.

The fourth assumption postulated that decreased *life space* will positively predict *intention to use*, which again was not the case in our study. More than half of the drivers in this study was either somewhat or totally dependent on someone to drive them, but only a minority indicated life space restrictions, as they did not travel further than “outside” their neighborhood. What is clear is that *intention to use* technology, especially as it pertains to autonomous vehicle technology, requires a different set of assumptions and preconditions to understand older adults' motivation to engage in such technologies. Thus, researchers need to focus on constructs that are much more telling of the indicators of older adults' successful engagement with AS.

Interestingly, our first regression model indicated, that from all the variables entered across the four models, only the first model was significant. Specifically, in this model older drivers' self-reported *driving difficulty* (*p* = 0.005) positively predicted their *intention to use* (Table 3). This is actually a very good sign that older drivers who are at risk, demonstrate as a group, the insight to want to use a safer mode (than driving) of transportation. However, this finding did not hold up as a significant predictor in the final SEM.

Finally, the fifth assumption postulated that predictor variables will singularly or cumulatively explain the eventual acceptance and adoption practices of older drivers—and hence we developed a conceptual model to explore the multi-variate relationships. Based on the PLS-SEM (Figure 2; Table 5), the results indicated that the path model can be used to generate hypotheses as discussed next.

**Table 5:** Predicting intention to use with driving difficulty and self-reported crashes and citations using backward stepwise selection.

**Variable**	**β**	***SE***	***t statistic***	***p***
Driving difficulty	0.162	0.077	2.109	0.037^**^
Crashes and/or citations	0.536	0.366	1.464	0.146

*β, path coefficient; SE, Standard Error*;

** *p < 0.05*.

First, increases in *technology readiness* are associated with an increase (*p* < 0.01) in *intention to use* (β = −0.247). Not surprising, this finding indicates a positive relationship between those who are ready to use technology and their intention to use the AS. Specifically, the items in the optimism domain indicate that new technology “contributes to better quality of life” (item 1), “gives more freedom of mobility” (item 2), “gives people more control over their daily lives” (item 3) and “makes me more productive in my personal life” (item 4). These items set the stage for planners, policy makers and industry partners to create opportunities for older adults to experience the benefits of the current AS technologies. Such experiences may positively impact the acceptance and adoption practices of older adults as they engage with AS, as early research is starting to illustrate (15, 25).

Second, when *perceived ease of use* increased, there was no change in *intention to use* (*p* > 0.05). It is not clear why *perceived ease of use* did not predict *intention to use*, especially because the items indicate that: interaction with the AV is clear (item #7), does not require a lot of mental effort (item #8), easy to use (item #9), and get the AV to do what one wants to do (item #10; Table 5). One potential reason for explaining the non-significant finding is that the older adults had only one exposure—and that occurred not in traffic, but in a bus depot, which may suggest that the true ease of use was not experienced in the context of daily life.

Third, a decrease in *barriers to AV acceptance* (meaning fewer barriers) was associated with a statistically significant increase (*p* < 0.001) in *intention to use* (β = −0.504). This finding has important implications for stakeholders of the AV industry. These stakeholders can make a significant contribution to reducing barriers for the older drivers pertaining to AS technology. For example, some of the AVUPS items underlying the *perceived barriers* include item # 5 “being suspicious of AV,” item #14 “require a lot of effort to use AV,” item # 26 “I believe AV will increase number of crashes,” and #28 “I feel hesitant about using AV” (36). Addressing these barriers, *via* education, exposure to the technology, demonstration rides, show-and-tell rides, workshops, roundtable discussions with drivers who had (vs. not had) exposure to AS, informational videos, and neighborhood trail rides, may go a long way in helping older adults be more prone to use the AS.

Fourth, when *life space* increased (meaning older adults ventured further away from their residences) there was no change in *intention to use* (*p* > 0.05). This result suggests that as older drivers are able to engage in a wider life space, that they do not have the need or intent to use the AS. City managers and industry partners can play an important role here in exposing older drivers to experience the benefits of using these AS technologies, while they are still independent (and driving), vs. having to wait until they can no longer drive—and are potentially more compromised, before exposing them to the AS technology.

Fifth, when *self-reported crashes and/or citations decreased*, when *driving exposure increased*, or when *driving difficulty* decreased (less driving difficulty), there was no change in *intention to use* AS (*p* > 0.05). Crash and/or citation involvement, that is not predictive of *intention to use*, is a bit perplexing to the research team. One phenomenon to consider is that the self-reported number of crashes may be underrepresented as we did not verify the self-reports with state or police reports of crashes and/or citations (78). On the other hand, less *driving difficulty* and increased *driving exposure* may indirectly indicate that older adults are more involved in their communities, which is very favorable. This may also suggest that the older adults may not necessarily have an *intention to use* the AS, as long as they can continue to be independent in their driving abilities, and venture in and outside of their communities.

When *cognition* increased (meaning better cognitive functioning), there was no significant change in *driving difficulty* or *intention to use* (*p* > 0.05). We were very surprised that cognition did not predict other sub-domains and/or *intention to use* in the model. Some of the reasons may include that our measure, TMT B, was just not adequately sensitive to detect actual changes; or that executive functions are not as important for *intention to use* as other domains of the cognitive construct. It is important to note that all participants were interested and willing to participate in the study, and thus had a baseline acceptance of riding in the AS. Finally, our sample had spectrum bias pertaining to cognition, as older drivers could only participate after meeting the MoCA criterion of <18 (out of 30). Other study limitations include self-selection bias due to COVID-19 pandemic, convenience sampling due to targeting one city area in FL, participants' interest to ride in the AS, and demographics that limit generalization to other diverse populations in the state, or across other states, in the U.S.

The strengths of our study, beyond what are already discussed in previous publications (15, 25, 36, 38, 39, 65, 79) pertain to revealing important exploratory information. Particularly, we have generated knowledge telling of the role of person factors (demographics, driving habits, cognition, life space), not previously examined in the AV and older driver literature. We have also demonstrated that the assessments or questionnaires, used to determine older drivers' declining driving abilities, are not necessarily predictive of their intention to use AS. Moreover, the PSL-SEM provides an important foundation to quantify core predictors of older driver performance, as cited in the literature, including their paths, coefficients and variance, for laying the founding for hypothesis generation and follow up studies in the older adult and AS industry.

Perhaps the greatest take home message of this study is the confirmation that city planners and policy makers, as well as industry partners and health care professionals, can play a role in the AS acceptance and adoption practices of older adults. Such actions may be proactive and overcome the current problem of intervening when older adults are experiencing too many comorbidities or declines, to actively learn and engage, in new transportation options, including the AS (80).

## Conclusion

This study examined personal predictors and aspects of technology readiness, ease of use, and barriers of intention to use AS. Although *cognition*, more specifically executive functions, are not identified as a predictor of such practices, *driving difficulty* did significantly predict *intention to use* AS in a linear model—but the results did not hold up in the final SEM. The PLS-SEM indicated that 47% of the variance in *intention to use* is explained by the predictor variables—even though only *technology readiness* and *barriers to AV acceptance* singularly predicted *intention to use*. Finally, we have identified opportunities for city managers, planners and policy makers, as well as industry partners, to institute proactive strategies to facilitate positive AS acceptance and adoption practices among older drivers.

## Data Availability Statement

The raw data supporting the conclusions of this article will be made available by the authors, without undue reservation.

## Ethics Statement

The University of Florida's Institutional Review Board (IRB#201801988) provided approval for the study and all participants consented to enroll and participate in the study. The patients/participants provided their written informed consent to participate in this study.

## Author Contributions

SC and VS: study conception and design. SH and JM: data collection and data input. JM: data management. JM and SC: analysis and interpretation of results. SC, JM, and VS: draft manuscript preparation. All authors reviewed the results and approved the final version of the manuscript.

## Funding

This research project (Project D2, #69A3551747104) received funding from the US Department of Transportation and the Southeastern Transportation Research, Innovation, Development, and Education Center.

## Conflict of Interest

The authors declare that the research was conducted in the absence of any commercial or financial relationships that could be construed as a potential conflict of interest.

## Publisher's Note

All claims expressed in this article are solely those of the authors and do not necessarily represent those of their affiliated organizations, or those of the publisher, the editors and the reviewers. Any product that may be evaluated in this article, or claim that may be made by its manufacturer, is not guaranteed or endorsed by the publisher.
