# Supplementary material for: Predicting Autonomous Shuttle Acceptance in Older Drivers Based on Technology Readiness/Use/Barriers, Life Space, Driving Habits, and Cognition
Source: Front Neurol. 2021 Dec 2;12:798762. doi: 10.3389/fneur.2021.798762 (PMC8674351; doi:10.3389/fneur.2021.798762)
Supplement: Supplementary file 1 [file Data_Sheet_1.docx]

**Supplementary Tables**

**Table S1.** Description of the variables

| # | Abbreviation | Transformation | type | Description |
| --- | --- | --- | --- | --- |
| 1 | Age | - | continuous | - |
| 2 | Intention to use | square root | continuous | AVUPS: Intention to Use subscale |
| 3 | Perceived Barriers | square root | continuous | AVUPS: Perceived Barriers subscale |
| 4 | Well-being | square root | continuous | AVUPS: Well-being subscale. |
| 5 | Acceptance | square root | continuous | Total Acceptance score of AVUPS |
| 6 | Crashes and Citations | - | binary | DHQ: Crashes and/or Citations. |
| 7 | Driving Exposure | - | continuous | DHQ: Driving exposure domain score |
| 8 | Driving Dependence | - | ordinal | DHQ: Dependence domain score |
| 9 | Driving Difficulty | square root | continuous | DHQ: Driving Difficulty domain score |
| 10 | Life Space | - | ordinal | LSQ total score |
| 11 | TMT B | - | continuous | Trail Making Test Part B score |

**Note:** AVUPS = Autonomous Vehicle User Perception Survey; DHQ = Driving Habits Questionnaire; LSQ = Life Space Questionnaire; TMT-B = Trail Making Test Part B.

**Table S2.** Backward stepwise selection for four multiple linear regressions.

| Model 1: Intention to Use ~ Age + TMT-B + Life Space + Driving Dependence + Driving Exposure + Driving Difficulty + Crashes and Citations | | | | |
| --- | --- | --- | --- | --- |
| Step | Variable removed | df | AIC | *R^2^* |
| 0 | Model 1 | 7 | 380.08 | 0.077 |
| 1 | Life Space | 1 | 378.08 | 0.077 |
| 2 | Driving Exposure | 1 | 376.10 | 0.076 |
| 3 | TMT B | 1 | 374.27 | 0.075 |
| 4 | Age | 1 | 372.56 | 0.072 |
| 5 | Driving Dependence | 1 | 371.74 | 0.062 |
|  |  |  |  |  |
| Model 2: Perceived Barriers ~ Age + TMT-B + Life Space + Driving Dependence +Driving Exposure + Driving Difficulty + Crashes and Citations | | | | |
| Step | Variable removed | df | AIC | *R^2^* |
| 0 | Model 2 | 7 | 390.74 | 0.098 |
| 1 | Driving Exposure | 1 | 389.09 | 0.095 |
| 2 | TMT B | 1 | 387.50 | 0.091 |
| 3 | Age | 1 | 386.64 | 0.081 |
| 4 | Driving Difficulty | 1 | 385.951 | 0.070 |
| 5 | Crashes and Citations | 1 | 385.899 | 0.052 |
|  |  |  |  |  |
| Model 3: Well-being ~ Age + TMT-B + Life Space + Driving Dependence + Driving Exposure + Driving Difficulty + Crashes and Citations | | | | |
| Step | Variable removed | df | AIC | *R^2^* |
| 0 | Model 3 | 7 | 423.02 | 0.071 |
| 1 | Life Space | 1 | 421.02 | 0.071 |
| 2 | Driving Difficulty | 1 | 419.03 | 0.071 |
| 3 | Driving Exposure | 1 | 417.59 | 0.066 |
| 4 | TMT B | 1 | 416.23 | 0.060 |
| 5 | Age | 1 | 415.15 | 0.052 |
|  |  |  |  |  |
| Model 4: Acceptance ~ Age + TMT-B + Life Space + Driving Dependence + Driving Exposure + Driving Difficulty + Crashes and Citations | | | | |
| Step | Variable removed | df | AIC | *R^2^* |
| 0 | Model 4 | 7 | 363.65 | 0.077 |
| 1 | Life Space | 1 | 361.71 | 0.076 |
| 2 | Driving Exposure | 1 | 359.85 | 0.075 |
| 3 | Age | 1 | 358.08 | 0.073 |
| 4 | TMT B | 1 | 356.37 | 0.070 |
| 5 | Driving Dependence | 1 | 355.80 | 0.058 |
